# Translation Validation, Software Adaptation, and Usability Testing of a Spanish Contraception Decision Tool: Protocol for a Mixed Methods Pilot Study

**DOI:** 10.2196/101800

**Published:** 2026-09-30

**Authors:** Minji Michelle Kang, Analay Perez, Alicia Brooks-Greisen, Daniela Burgos, Raphaela Barrios, Desiree Virginia Fermin Olivares, Andrea Perez Mukdsi, Mikel Llanes, Murphy Van Sparrentak, Cynthia Wynn, Justine P Wu

**Affiliations:** 1 Department of Family Medicine, University of Michigan, North Campus Research Complex, 2800 Plymouth Rd. MM Family Medicine B16, Suite 400S, Ann Arbor, MI, 48109-2800, United States, 1 9898590950; 2 DARTNet Institute, Aurora, CO, United States; 3 University of Michigan College of Pharmacy, Ann Arbor, MI, United States; 4 Department of World Languages and Cultures, Georgia State University, Atlanta, GA, United States; 5 Women's, Gender, and Sexuality Studies, Georgia State University, Atlanta, GA, United States; 6 Department of Obstetrics and Gynecology, University of Michigan, Ann Arbor, MI, United States

**Keywords:** mixed methods, iterative convergent design, translation, contraceptive decision tool, chronic disease, birth control, contraception, decision-making

## Abstract

**Background:**

Contraceptive care is complex, especially for individuals with health conditions (eg, diabetes, depression, and cardiovascular disease) who must consider how and to what extent their condition may be affected by contraception or vice versa. My Health, My Choice (MHMC) is a novel, web-based contraceptive decision support tool designed in English for patients with health conditions and is currently being tested in a cluster randomized controlled trial in the United States. MHMC is not available in Spanish, creating linguistic barriers to trial participation for those who prefer to read and learn in Spanish.

**Objective:**

This protocol describes a 3-stage process to create a medically accurate, linguistically and culturally acceptable Spanish version of MHMC called *Mi salud, mi decisión*.

**Methods:**

The pilot study combines an iterative, convergent mixed methods design with an innovative translation process and user experience methodology. In stage 1 (translation validation), a bilingual 8-person translation team will conduct the Team-Driven, Person-Centered Translation Process, a 7-step innovative process developed for this study: (1) resource and team preparation; (2) forward translations; (3) synthesis of translations; (4) back translation; (5) consensus (harmonizing) of translations; (6) review and proofreading; and (7) pretest or pilot of the translated adaptation. In stage 2 (software adaptation), we will work with a professional software team to internationalize MHMC (prepare it to technically support multiple languages) and then localize MHMC (prepare it for a specific language or region) for use in Spanish. In stage 3, we will conduct usability testing with a nonprobability sample of 12 to 20 Spanish-speaking individuals (aged 18‐49, who can get pregnant and want contraceptive decision support) who vary in reading levels and Spanish dialects. Quantitative usability data will be collected via electronic survey with an adapted version of the Spanish System Usability Scale (SUS). Qualitative data will be collected via “think aloud” cognitive interviews during which participants navigate the tool in real-time. We will conduct iterative cycles of rapid qualitative analysis and software revisions to identify and resolve user-interface problems and improve the Spanish text for medical accuracy, linguistic acceptability, and cultural acceptability. Usability testing will stop when data no longer yield substantive feedback.

**Results:**

As of September 7, 2026, we have enrolled 5 participants to date and anticipate completion of the pilot by November 1, 2026. We anticipate completion of qualitative analysis by December 2026 and publication of results in 2027. The final product, *Mi salud, mi decisión*, will be a contraceptive decision tool in Spanish with semantic equivalence (similar meanings of words) and conceptual equivalence (similar meanings of concepts) to the original English MHMC.

**Conclusions:**

This protocol describes translation validation, software adaptation, and usability testing of a novel web-based contraceptive decision tool in Spanish. Upon completion of this project, *Mi salud, mi decisión* will be ready for efficacy testing in a cluster randomized controlled trial.

## Introduction

Contraceptive decision-making can be a complex process for many people when planning their reproductive goals, including pregnancy prevention [1,2]. There are 18 contraceptive methods (hereafter referred to as “methods”), which include nonprescription and behavioral methods, prescription methods, contraceptive devices, and permanent contraception (tubal surgeries and vasectomy) [3]. Not only is there an overwhelming number of methods to choose from, but methods differ in frequency of use, failure rate, side effects, benefits, and user experience (UX) [3].

Contraceptive decisions become more complicated when people also have acute or chronic health conditions to take into consideration [4]. To make informed decisions, they should understand the impact that a specific method could have on their health condition(s) and medications, and how their health conditions and medications may affect their reproductive health (fertility and pregnancy) [5,6]. However, studies have shown that this population receives inconsistent or incomplete contraceptive counseling [7-11]. For example, a regional study of the United States found that primary care patients aged 18 to 50 years who reported poorer health had decreased odds of receiving comprehensive contraceptive counseling at their visit compared with patients who reported better health [11]. The Centers for Disease Control and Prevention (CDC) US Medical Eligibility Criteria (US MEC) is a clinical tool that categorizes contraceptive methods by medical risk category for more than 60 health conditions and patient characteristics [12]. However, the recommendations are intended to be a resource for health care providers rather than individual patients [12]. At this time, no comprehensive resource exists that educates patients with health conditions in layperson language and user-friendly format.

To address this unmet need, we designed a novel, theory-informed web-based tool called My Health, My Choice (MHMC) [4,13]. Contraceptive decision tools are helpful resources that can support patients in this selection process through method education in the context of personal needs, values, and preferences [14]. This interactive tool provides contraceptive method education within the context of a person’s health condition, medications, personal characteristics, and preferences [4]. MHMC is currently being tested in an ongoing National Institutes of Health–funded (NCT07075536) randomized controlled trial (RCT) [4]. However, to date, Spanish-speaking patients are excluded from the trial because there is no Spanish version of MHMC.

For the purposes of this study, we focus on “Spanish-speaking individuals” and acknowledge that this term includes many races and ethnicities. When we refer to specific racial or ethnic groups, such as Hispanics or Latino(a)s, we will use the same terms that the authors used in the original articles.

The growing population of Spanish-speaking patients in the United States [15] should have access to accurate and culturally acceptable resources to make informed health decisions. As of 2021, US Spanish speakers represent 63% of the population with limited English proficiency seeking health care [16]. Spanish-speaking Hispanics report worse health status and access to care than English-speaking Hispanics, 39% vs 17% in fair or poor health, respectively, in the United States [17]. Compared to non-Hispanic White Americans, Hispanic Americans are more likely to be obese, develop diabetes, and have high blood pressure [18]. Spanish-speaking individuals in the United States experience inequities in reproductive health care quality and access [19,20]. In 2024, a nationally representative survey found that US patients with lower English proficiency are less likely to receive high-quality contraceptive care (27%) than their English-speaking counterparts (52%) [20-22]. Young Latina women are more likely to discontinue or not use any contraceptive method compared to their non-Hispanic White counterparts [23-25]. Historically, a lack of resources and tools in a patient’s preferred language creates significant barriers to comprehensive health care [26]. For example, a systematic review about the implications of language barriers found that lack of resources in preferred languages led to miscommunication between provider and patient, lower patient satisfaction, and a significant decrease in the perceived quality of health care delivery [26]. In reproductive health, studies have found that patients with language barriers face systems of oppression that drive inequities such as forced sterilization, obstetric trauma, and lower rates of gynecologic cancer screenings [27].

Compounding the lack of translated resources, language translation processes have been criticized for inaccurate or culturally insensitive translations of research and intervention materials [28]. Researchers are often not involved in the translation process, outsource translation to others, and have little knowledge of best practices in translation, such as linguistic accuracy and cultural acceptance [28]. These oversights have led to inaccurate and culturally inappropriate “word-for-word” translations of data collection instruments and interventions that fail to convey the original intent of the source text, further contributing to health care disparities [28,29].

In this protocol paper, we describe the creation of a Spanish version of MHMC, called *Mi salud, mi decisión*, a contraceptive decision support tool for Spanish-speaking patients with health conditions.

## Methods

### Study Overview

This study consists of 3 stages, each with a specific objective and expected product (Figure 1). Stage 1 (translation validation) objective is to rigorously translate all English content into clear, medically accurate, and linguistically and culturally acceptable Spanish; the expected product is a Spanish blueprint (S-blueprint) for all Spanish text. Stage 2 (software adaptation) objective is to internationalize (ie, prepare the tool’s software to technically support multiple languages) and localize (ie, prepare the tool specifically for use in Spanish); the expected product is a functional prototype of *Mi salud, mi decisión* ready for usability testing. Stage 3 (usability testing) objective is to elicit actionable feedback from potential end users and revise the software to resolve identified problems; the expected product is an optimized version of *Mi salud, mi decisión* ready for efficacy testing in an RCT.

**Figure 1.**
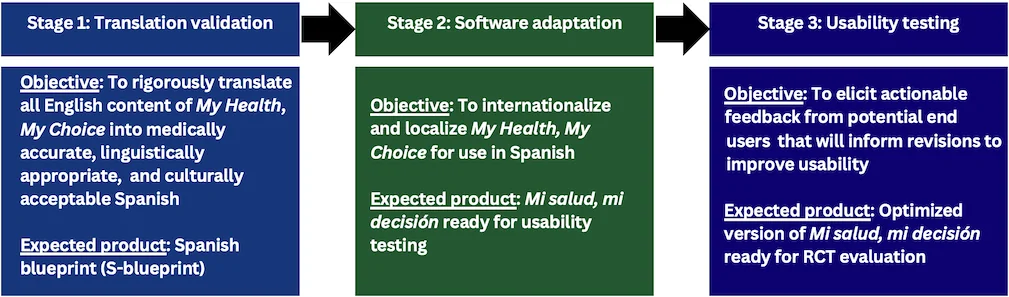
Three-stage process for creating a Spanish contraceptive decision tool, *Mi salud, mi decisión*. RCT: randomized controlled trial.

### The Original Intervention in English: MHMC

MHMC is a theory-informed decision support tool designed to help patients understand their contraceptive options in the context of their health conditions, medications, and personal characteristics. MHMC is compatible with mobile phones (iOS or Android), laptops, desktop computers, and tablets. A detailed description of the intervention design has been previously described [4]. MHMC has 3 components: an administrative interface, a patient interface, and a clinician interface. The administrative interface is a backend system to manage and update content. In the patient interface, the user indicates which of 63 health conditions (eg, diabetes and hypertension) and characteristics (eg, smoking), and 50 medications associated with fetal defects and/or interacts with hormonal contraception (eg, warfarin and lamotrigine) apply to them. The tool’s logic rules then deliver tailored contraception education that uses sixth to eighth grade-level language to explain the risks and benefits of different methods in the context of the user’s health condition, medications, and/or personal characteristics. Personal preferences about menses and future pregnancy desires (if any) are elicited. A Notebook feature allows patients to write down questions or jot notes in preparation for a discussion with their clinician. The tool displays the contraceptive methods grouped by medical risk category per evidence-based guidelines by the CDC US MEC [12]. Patients are shown methods grouped by traffic-light color scheme and text for layperson comprehension: categories 1 and 2 represent “Safe. Use any of these” (green bar); category 3 represents “Caution. These methods may be risky for you. Use only if the benefits outweigh the risks to you. Talk to your clinician.” (yellow bar); and category 4 represents “Stop. Do not use these.” (red bar). Interactive features allow patients to compare methods side by side, as well as “favorite” methods of interest. A Birth Control Summary feature synthesizes the patient’s responses, including their contraceptive “favorite” and Notebook entries.

A board-certified family physician who has expertise in contraceptive care and best practices in patient communication (JPW) wrote all patient education content. A scientific advisory panel that included 3 expert authors of the CDC US MEC guidelines and 6 physician experts conducted an independent review of all content and provided feedback.

The clinician interface is designed to help clinicians counsel their patients based on the information already provided by the patient. Just prior to or during the visit, clinicians can review the “My Birth Control Summary” via a secure link on the clinician interface. This summary minimizes clinician burden and ensures discussions are driven by tailored patient concerns and priorities. Clinicians are also provided with curated snapshots and text descriptions of the US MEC that highlight conditions, medications, and/or characteristics relevant to the patient. A full list of conditions, characteristics, and medications can be found in Multimedia Appendix 1.

### Ethical Considerations

Survey data will be collected via University of Michigan (UM) REDCap (version 16.0.39; Vanderbilt University), a secure web application for research data collection and management hosted at UM via National Institutes of Health grant UM1TR004404 [30]. Interviews will be conducted and recorded via the UM Zoom platform. Upon completion of each interview, the research assistant (RA) will upload the video recording file labeled by participant ID only (and no personal identifiers) to the UM Dropbox. No personal identifiers will be stored in UM Dropbox. After confirming that the recording has been successfully uploaded, the RA will delete the original recording from the UM Zoom source file. UM REDCap, UM Zoom platform, and UM Dropbox are all password-secured and HIPAA (Health Insurance Portability and Accountability Act)-compliant.

A RA will email individuals who express interest in the study to schedule an eligibility screening by phone or videoconference on the UM Zoom platform. During the phone or video call, the RA will review the consent form and give the individual an opportunity to ask questions. If eligible and interested, the individual will verbally provide prospective agreement to study participation. The participant will be scheduled for an interview via UM Zoom videoconference.

This study protocol was approved for exempt status by the UM Institutional Review Board (HUM00285806). We will register this pilot study prior to analysis [31].

### Stage 1: Translation Validation

#### Development of the Team-Driven, Person-Centered Translation Process (TPTP)

Our translation process is informed by the Participatory and Iterative Process Framework for Language Adaptation (PIPFLA), guidelines created by the European Organization for Research and Treatment of Cancer (EORTC), and best practices in translation [32-34]. The PIPFLA is an 11-step process developed in response to sparse literature regarding best practices for language translation of evidence-based health interventions [29]. The EORTC guidelines outline a rigorous 5-step translation procedure that promotes accurate and contextually appropriate translations inclusive of various linguistic and cultural regions for data collection instruments [28]. Both guidelines involve forward and back translation and a form of synthesis (harmonizing translations or finding consensus about translations among a team). Forward translation is the process of direct translation of documents in their original source language to the target language [35]. Back translation is the process of translating content from a target language back to its original source language [36]; for example, in this study, the Spanish content will be back translated into English.

Our adaptation, the TPTP, is a 7-step procedure that leverages the strengths of PIPFLA and the EORTC guidelines. To develop TPTP, we compared PIPFLA and EORTC side by side, selected steps that we deemed most critical and actionable, removed steps that were redundant or could be combined with other steps, and expanded upon steps to improve generalizability and replicability. Through this process, we retained all 5 steps of the EORTC guidelines, which are conceptually similar to steps 2 to 5 of the PIPFLA. Steps 6 to 9 of PIPFLA outline 2 cycles of harmonization and expert review; we condensed these into 2 steps (steps 5 and 6). Finally, we renamed and expanded upon PIPFLA’s step 1 “preparation” and step 10 “proofreading” as TPTP step 1 “resource and team preparation” and step 6 “review and proofreading,” respectively. Textbox 1 displays the 11-step PIPFLA, the 5-step EORTC, and our newly created 7-step TPTP.

Below, we describe each step of the TPTP and the criteria for success that must be met before moving on to the next step, where relevant. Figure 2 is a visual diagram of the TPTP.

Textbox 1. **Team-Driven, Person-Centered Translation Process (TPTP) steps adopted from the Participatory Iterative Process Framework for Language Adaptation (PIPFLA) and European Organization for Research and Treatment of Cancer (EORTC) guidelines.**

**PIPFLA**

- Preparation (adopted in the TPTP)
- Forward translation (adopted in the TPTP)
- Back translation (adopted in the TPTP)
- Review of back translation (adopted in the TPTP)
- Harmonizing (adopted in the TPTP)
- Expert review
- Harmonizing
- Expert review
- Harmonizing
- Proofreading (adopted in the TPTP)
- Creating final adaptation (adopted in the TPTP)

**EORTC guidelines**

- Two forward translations (adopted in the TPTP)
- Synthesis of translations (adopted in the TPTP)
- Back translation (adopted in the TPTP)
- Consensus of translations by committee or team (adopted in the TPTP)
- Pretest or pilot of final adaptation (adopted in the TPTP)

**TPTP**

- Resource and team preparation
- Two forward translations
- Synthesis of translations
- Back translation
- Consensus of translations (harmonizing) by committee or team
- Review and proofreading
- Pretest or pilot of final adaptation

**Figure 2.**
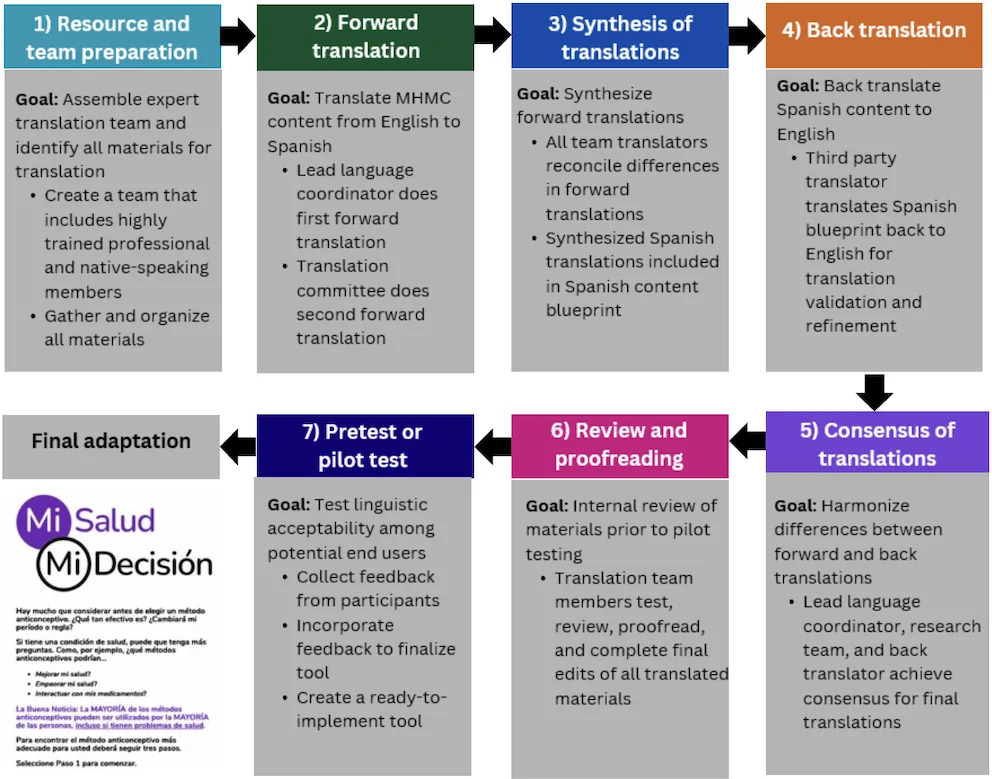
Team-Driven, Person-Centered Translation Process (TPTP) informed by evidence-based frameworks. MHMC: My Health, My Choice.

#### Resource and Team Preparation

In the original PIPFLA, part of step 1 (preparation) is “(getting) permission from developers and publishers,” which is not relevant to this study since we developed the original source materials. To broaden the application of this step, we renamed it “resource preparation” and define it as identifying and organizing all source materials into a user-friendly format with version control and track changes, and, if relevant, obtaining permissions to translate materials developed by others. For this study, we consolidated our source materials into a single 253-page shared Word document that is only accessible to team members, referred to as the English blueprint (E-blueprint).

Step 1 of the PIPFLA also calls for “a team of experts and translators” without specific guidance about the team composition. Best practices in the translation literature advise a team-based approach with professional bilingual translators and people who share characteristics of the intended users [32-34]. While a team could consist of only 2 people (one for back translation and one for forward translation), we advise a larger team, if feasible, with members from communities similar to those of the intended end users. We anticipate that a carefully selected team of translators, coupled with intentional team-building processes, will generate nuanced considerations of Spanish dialects, colloquialisms, and commonly used medical and health terms.

Our translation team consists of 8 highly qualified individuals who reflect a wide range of Spanish speakers in the United States: 2 native Spanish-speaking RAs (DB and RB, who identify as Puerto Rican and Bolivian, respectively); a translation coordinator (AB-G), a nonnative Spanish speaker with formal education and more than 15 years of experience with Spanish and English translation and interpretation in research and medical settings; a bilingual medical expert, a native Spanish-speaking Cuban doctor (ML), and a professional translation committee of 4 female native speakers (Paraguayan, Argentinian, Guatemalan, and Mexican), 3 of whom are professional translators and one of whom is a small business owner and community mobilizer. To ensure that the final translation reflects the contributions of all translation team members, we have prepared team-building strategies. First, we will implement a round-robin format so everyone has a chance to speak during weekly meetings. We will open each meeting with a “Rose, Bud, Thorn” (ie, positives, opportunities, and challenges) self-reflection exercise to create a sense of belonging, trust, and group cohesion [37]. To promote information-sharing and transparency in decision-making, all team members will be included on email communications, given access to source documents with track changes and comments, and encouraged to review and add edits and comments.

Throughout all 7 steps of TPTP, the translation team will make decisions needed to localize MHMC specifically for Spanish, which will have implications for software adaptations in step 3. Examples include how to manage gendered nouns and adjectives commonly used in Spanish, and the use of formal vs informal language.

#### Forward Translations

Step 2 involves 2 independent forward translations similar to the EORTC guidelines. Two forward translations completed by different teams may reduce the burden of subsequent harmonization and revisions by including multiple experts earlier in the process. The translation coordinator will conduct the first forward translation of the entire E-blueprint and use ChatGPT (OpenAI) as a drafting aid only. A 2023 study found that machine-generated translations can accelerate and improve the accuracy of translation as long as translation processes are combined with human quality checks [38]. The translation committee will simultaneously and independently conduct a second forward translation. Each translation committee member will be assigned specific portions of the E-blueprint and tasked with translating assigned sections based on their own interpretations and native Spanish dialect. Consistent with the principles of cultural translation, the committee translators will serve as cultural brokers to ensure that terminology and phrasing are meaningful and acceptable across diverse Latin American Spanish-speaking groups with varying regional and sociocultural linguistic registers.

#### Synthesis of Translations

The goal of step 3 is to create a single harmonized forward translation, called the Spanish blueprint (S-blueprint). The translation coordinator and the translation committee will meet via web conference and communicate asynchronously to identify and reconcile differences between the 2 forward translations. When multiple acceptable variants for terms exist, the translation committee will select terms that are judged to be the most widely understood across multiple dialects. This step will be considered complete when all discrepancies have been reconciled by team consensus.

Our bilingual medical expert (ML) will review and edit the entire S-blueprint for medical accuracy, patient safety, and consistency with the intent of the original text, particularly the explanations of the US MEC risk categories and potential method-related health risks.

#### Back Translation

Step 4 of the TPTP will be a back-translation process of the S-blueprint created in step 3. To ensure a valid back translation, we will not share any of the E-blueprint with the back translator (ML). For practical purposes, ML will not back translate the entire 253 pages of content and will instead selectively back translate text deemed critical by the team and the principal investigator (JPW) for usability (eg, login, homepage, buttons, and introductions), patient safety, and education about contraceptive method features, including pros, cons, and medical risk categories. ML will conduct the back translation using a worksheet that includes Spanish translations of selected content from the S-blueprint with matching blank columns for back translations to English and relevant comments. A snippet of this worksheet with hypothetical examples can be seen in Multimedia Appendix 2.

#### Consensus (Harmonizing) of Translations

Step 5 of the TPTP mirrors the back translation review step in both the PIPFLA and EORTC guidelines. The translation coordinator and a member of the research team (MMK) will review the back translation worksheet and address inconsistencies and comments, directly consulting the back translator as needed. The entire translation team will then review and provide feedback on the revised back-translation worksheet, and differences will be reconciled by MMK and the translation coordinator. The process will repeat until the entire translation team reaches consensus that semantic and conceptual equivalence with the E-blueprint’s intent and meaning has been achieved.

#### Review and Proofreading

Step 6 of the TPTP adopts PIPFLA’s tenth step by ensuring that all harmonized materials are reviewed and proofread before testing. Translation team members DB and RB will conduct a final review of translations from the perspective of an end user. Any issues identified during review and proofreading will be addressed via adjudication with the translation coordinator, as needed, prior to step 7.

#### Pilot or Pretest

Step 7 is adapted from the pilot or pretest step of the EORTC guidelines. This step involves testing among potential end users, which is discussed in stage 3.

### Stage 2: Software Adaptation

Software adaptation will proceed in 2 steps: internationalization and localization. Internationalization is the process of developing new software or adapting existing software to technically support multiple languages without the need for significant re-engineering later [39]. Localization refers to the process of preparing the software to be used in a specific language or region [39].

Alfa Jango, the professional software team that helped create MHMC’s software design, will perform internationalization. This process includes but is not limited to the following: implementing automatic detection of the preferred language on the user’s device; redesigning graphics and buttons to accommodate other languages, including those that read right-to-left (eg, Arabic); and ensuring previously hard-coded text is properly extracted into the translation system. Alfa Jango will also meet weekly with the research team to identify additional requirements to localize MHMC specifically for Spanish (eg, configurations to enable the use of Spanish-specific accent marks).

When internationalization is complete, the translation team RAs will import the entire S-blueprint into the administrative platform. Throughout this process, the translation team will conduct quality checks by using the tool as an end user would, according to assigned mock patient profiles. Technical errors and features that are not working as intended will be resolved by Alfa Jango. We will proceed to stage 3 when we cannot identify any further errors or feature-related problems.

### Stage 3: Usability Testing

#### Stage 3 Design Overview

Stage 3 involves pilot testing among potential end users through UX design methodology. UX design is a well-established methodology for developing interventions and involves the end users throughout development and testing [40]. The methodology is an iterative process that involves multiple rounds of testing, eliciting feedback, making necessary changes, and repeating the cycle until an acceptable product is formed [40], resulting in higher quality end products [41].

Alwashmi et al [42] advance the rigor of usability testing by proposing a hybrid methodology involving mixed methods research. Their novel mixed methods design for mobile health usability testing integrates the iterative testing process of UX design and a convergent mixed methods design. A convergent mixed methods design involves the simultaneous data collection and analysis of quantitative and qualitative research [43]. This pilot study will involve multiple rounds of testing by integrating quantitative data collected via electronic surveys and qualitative data collected via video conference interviews. Figure 3 illustrates our adaptation of the iterative convergent mixed methods design.

**Figure 3.**
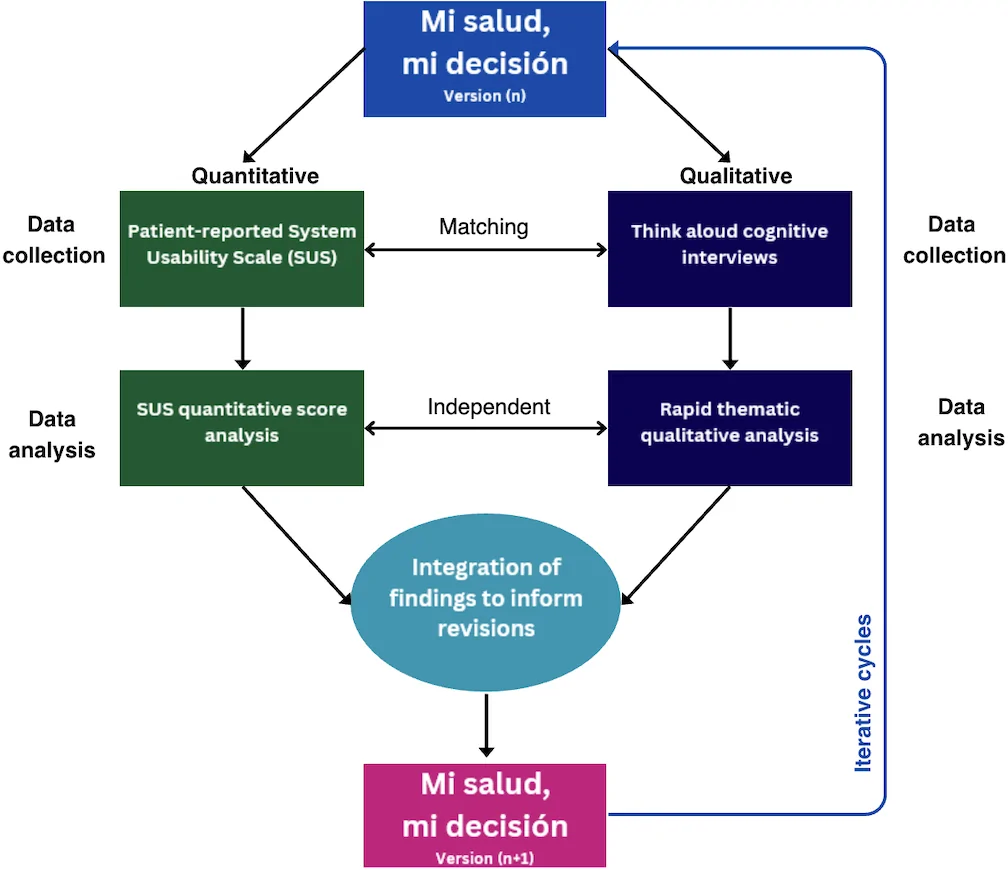
*Mi salud, mi decisión* usability testing iterative, convergent mixed methods design.

#### Participant Eligibility, Recruitment, Consent, and Enrollment

Eligible participants must (1) be aged 18 to 49; (2) be assigned female sex at birth (any gender identity acceptable); (3) have access to a mobile phone, computer, or tablet with internet access; (4) be fertile (have an intact uterus and at least one ovary, being premenopausal, having never had permanent contraception surgery, and having no known medical reasons for infertility); (5) have at least one index health condition and/or take an eligible medication; (6) not be currently pregnant; (7) have the desire to start, switch, or add a contraceptive method for the purpose of preventing pregnancy; and (8) be able to read in Spanish and use it as a preferred language. Participant exclusion criteria include people who are trying to become pregnant in the next 12 months and/or who are presumed infertile for any reason.

We will use 3 strategies for participant recruitment. First, we will post the study on the UM Health Research website, which disseminates study opportunities to over 100,000 people nationally who have indicated interest in health research. Second, we will post flyers in local establishments frequented by Spanish-speakers and in public spaces. Third, if needed, we will use word-of-mouth referrals from community organizations that serve Spanish-speaking people and partner with UM.

#### Collection and Analysis

##### Quantitative Data Collection

We will send participants a web-link to test *Mi salud, mi decisión* with a unique identification code and ask them to interact with the tool as if they were preparing to visit their clinician for contraceptive counseling. After using the tool, we will ask them to complete the Spanish System Usability Scale (SUS) and a demographics survey via a unique link to a REDCap survey. Demographics include age, gender identity, sexual orientation, race, ethnicity, insurance status, education level, weight, health history, pregnancy and sexual history, and recent contraceptive use. The Spanish SUS is a 10-item survey and a reliable and validated scale of perceived usability across a range of technology platforms (eg, websites and smartphones) [44]. The user rates each item from 1 (“strongly disagree” or “totalmente en desacuerdo”) to 5 (“strongly agree” or “totalmente de acuerdo”); the total SUS score range is 0 to 100, with higher scores indicating greater usability. We modified the scale’s language to be contextually relevant to the study (Multimedia Appendix 3). For example, the item that asks, “Me gustaría usar esta herramienta frecuentemente” (“I think that I would like to use this system frequently”) was changed to “Pienso que el uso de esta herramienta podría ayudarme a elegir el método anticonceptivo indicado para mi” (“I think that I would use this tool to help me choose a birth control method that’s best for me”).

##### Quantitative Data Analysis

We will conduct descriptive statistics to characterize our sample demographics and the distribution and measures of central tendency for the total SUS scores. We will also examine individual SUS scores for each of the 10 scale items [44].

##### Qualitative Data Collection and Quality Checks

Two bilingual team members, AB-G (the translation coordinator) and DVFO, will conduct all semistructured interviews, taking turns leading the interview or taking notes. Given our need for timely and actionable data to inform iterative cycles of software revisions, we will selectively transcribe parts of the interviews verbatim, but not the whole interviews. Prior research has shown that selectively transcribed interviews, generated through structured protocols and validity checks, can yield contextually-rich data and reduce the delays and costs of full verbatim transcription [45,46].

To ensure consistency across interviews, we developed a structured template to guide systematic note-taking. The template is organized to mirror the user’s stepwise journey interacting with the tool, eliciting the user’s explanations for their SUS item ratings, and feedback on linguistic and cultural acceptability. The template also includes sections to document team debriefs and user-identified problems with the tool’s interface, content, and features. To select parts of the interview for verbatim transcription, the interviewers will prioritize the following: highly personal and rich descriptions related to the research topic (ie, the quality of the Spanish translation, reactions to the contraceptive and reproductive health education, and opinions about the tool’s usability); uncommon experiences or unique opinions; and new insights about the tool or the study’s research topics. In contrast, interview sections that should be summarized with paraphrasing and do not require verbatim transcription include brief utterances; comments unrelated to the research topics (eg, the weather, opinions about nonreproductive health or contraceptive care); and general descriptions of the tool’s features or content without specific feedback.

Prior to starting the interview, the interviewer will confirm the individual’s agreement to participate and to be video-recorded. The participant will then be asked to open their unique web link to *Mi salud, mi decisión* and share their screen so that AB-G and DVFO can observe them using the tool in real-time. Using a semistructured interview guide, AB-G or DVFO will elicit participant feedback by asking them to expand upon their SUS responses. For items rated 3 or below, participants will be asked to explain why they did not feel that the tool performed well in that specific area or topic of usability. For items rated 4 or 5, participants will be asked to explain why they felt the tool did perform well in that specific area or topic of usability. Participants will be encouraged to suggest improvements to the tool in each domain of the SUS. The interviewer will also assess linguistic and cultural acceptability by asking participants to comment on terms or words and how they might change the language based on their own experiences.

We have built-in quality checks for accuracy and internal validity. Right after the interview, AB-G and DVFO will debrief to arrive at a shared understanding of the participant’s responses, select which parts of the interview to transcribe verbatim, and document user-interface problems in detail. Using the video recording, they will verbatim transcribe selected interview parts, as previously selected, in the template. Two bilingual RAs (DB and RB) will then review the completed template and the video recording for accuracy, edit as needed, and add more verbatim transcriptions as they deem necessary. DB and RB will then back translate the entire interview template into English; AB-G and DVFO will review and edit the English back translation for linguistic and conceptual equivalence. As a final audit, JPW, the senior investigator with contraceptive, qualitative, and mixed methods expertise, will review each template in English and highlight statements that require further review and discussion during team meetings.

##### Qualitative Data Analysis

We will modify the rapid and accelerated data reduction technique [47], a structured qualitative analysis approach well-suited for mixed methods usability testing. It has five steps: (1) format transcripts in a similar manner (for our study, standardized interview templates as described above), (2) incorporate all templates into an all-inclusive data sheet and prepare for phase 1 data reduction, (3) engage in phase 1 reduction by reducing nonrelevant text that fails to address the research question(s) and create a phase 2 data reduction table, (4) further reduce and refine the phase 2 table to develop a phase 3 condensed table that includes data most relevant to the research question(s), and (5) create project deliverables (ie, revisions for the tool). The analysis team (AG-B, DVFO, RB, DB, and MMK), led by JPW, will meet weekly to review each interview template (English version with in-vivo Spanish quotes), discuss findings, resolve differences, and update the interview guide, as needed, based upon emerging findings.

### Mixed Methods Analysis

Quantitative and qualitative data will be examined separately (a mixed methods integration strategy called *independent analysis*) before integrating for feedback incorporation. We will intentionally integrate participants’ quantitative responses to the usability of the tool with the qualitative themes (a mixed methods integration strategy called *matching*). A joint display, or visual representation of quantitative, qualitative, and mixed methods findings, will be used to map qualitative feedback to SUS scores for each item and the total score. This type of display is a common integration technique for interpreting both quantitative and qualitative findings [48]. The joint display will present meta-inferences (ie, integrated conclusions) that will inform feasible suggestions or improvements (qualitative data) based on low SUS scores (quantitative data).

### Application of Qualitative and Mixed Methods Findings

Each cycle of qualitative and mixed methods analysis will identify high priority revisions to the tool, defined as language changes to improve medical accuracy, linguistic acceptability, or cultural acceptability, and software updates to resolve user-interface errors. To create a Spanish version of the tool that can be evaluated alongside the English version in an RCT, we will not implement any new features or educational content at this time. However, we anticipate that participants will share ideas for improvement; these data will be analyzed and published to inform future studies.

After software revisions have been implemented and quality-tested, we will conduct another cycle of data collection with new participants. This process will repeat until we have reached data saturation [49,50], the point at which we no longer identify any high-priority revisions as described above. We estimate this will occur after 12 to 20 participants (3‐4 cycles of 4‐5 participants per cycle).

## Results

As of September 7, 2026, we have enrolled 5 participants to date and anticipate completion of the pilot by November 1, 2026. We anticipate completion of qualitative analysis by December 2026 and publication of results in 2027. This pilot study will result in a semantically and conceptually equivalent Spanish version of MHMC, ready to be evaluated in an RCT.

## Discussion

To date, MHMC is the first decision-support tool focusing on contraceptive decision support for people with health conditions, and *Mi salud, mi decisión* will be the first available in Spanish. This pilot study also introduces our innovative translation process, TPTP, which adopts the strengths of 2 prior approaches into 1 protocol with a team-based approach and user-centered testing. TPTP offers a new framework to efficiently and accurately translate behavioral health interventions and test translations among potential end users to ensure linguistic and cultural acceptability before deployment. Another strength of this pilot study is the integration of mixed methods and UX design. Using an iterative convergent, mixed methods design generates unique and robust insights into usability and acceptability that singular methodologies could not produce. UX design centers on potential end users and creates a product of higher quality. Together, these strengths position *Mi salud, mi decisión* as both scientifically rigorous and acceptable to Spanish-speaking patients with health conditions, increasing the likelihood that the tool will be usable, acceptable, and implementable in clinical settings.

There are study limitations. First, this pilot will result in changes to improve the tool’s medical accuracy and linguistic acceptability, but not its content or design. It is possible that there are content or tool design features that may be desired by this population that have not been identified by English-speakers in the original pilot of MHMC. Content or design refinements will be prioritized for incorporation in a subsequent update once the current RCT is complete. Second, this study may exclude people with lower levels of digital literacy or who cannot access a digital device. Future studies could offer technical support as part of an intervention bundle, and they could purposefully measure digital literacy and offer additional support (eg, guided onboarding, brief training, or assisted-use sessions) to better understand participants’ experiences.

We expect the findings of this pilot to increase accessibility for patients who prefer to read and learn about contraception in Spanish by optimizing linguistic and cultural acceptability to best represent diverse dialects and cultures.

## Supplementary material

10.2196/101800 Multimedia Appendix 1 My Health, My Choice: list of index health conditions, characteristics, and medications.

10.2196/101800 Multimedia Appendix 2 *Mi salud, mi decisión* back-translation worksheet with examples.

10.2196/101800 Multimedia Appendix 3 Modified Spanish System Usability Scale for *Mi salud, mi decisión* usability testing.

10.2196/101800 Peer Review Report 1 Peer review report by: CMGC - Clinical Management in General Care Settings Study Section, Eunice Kennedy Shriver National Institute of Child Health and Human Development (National Institutes of Health, USA).

## Abbreviations

CDC: Centers for Disease Control and Prevention
EORTC: European Organization for Research and Treatment of Cancer
HIPAA: Health Insurance Portability and Accountability Act
MHMC: My Health, My Choice
PIPFLA: Participatory and Iterative Process Framework for Language Adaptation
RA: research assistant
RCT: randomized controlled trial
SUS: System Usability Scale
TPTP: Team-Driven, Person-Centered Translation Process
UM: University of Michigan
US MEC: US Medical Eligibility Criteria
UX: user experience
