# Supplementary material for: Translation Validation, Software Adaptation, and Usability Testing of a Spanish Contraception Decision Tool: Protocol for a Mixed Methods Pilot Study
Source: JMIR Res Protoc. 2026 Sep 30;15:e101800. doi: 10.2196/101800 (PMC13626405; doi:10.2196/101800)
Supplement: Supplementary file 2 [file resprot-v15-e101800-s002.docx]

Back Translation Worksheet with Examples

E-blueprint (Original English MHMC Content)	Forward Translation (English->Spanish)	Back Translation (Spanish -> English)	Notes from Back Translator
**Hypothetical Example**			
Fact 2: The progestin implant (Nexplanon) is very safe for people with health conditions. The implant is a thin single rod (matchstick size) that is placed in the upper arm after the skin has been numbed.<br>Like IUDs, the implant can be used safely by almost all people, even those with serious health problems. The implant is 99% effective and lasts up to 5 years. You can ask your health care provider to take it out anytime earlier. The implant does not affect your fertility (your natural ability to get pregnant).	Dato 2: El implante de progestina (Nexplanon) es muy seguro para personas con problemas de salud. El implante es una varilla delgada (del tamaño de una cerilla) que se coloca en la parte superior del brazo después de adormecer la piel.<br>Al igual que los DIU, el implante puede ser utilizado con seguridad por casi todas las personas, incluso aquellas con problemas de salud graves. El implante tiene una eficacia del 99 % y dura hasta 5 años. Puede pedirle a su proveedor de atención médica que se lo retire en cualquier momento antes. El implante no afecta a su fertilidad (su capacidad natural para quedarse embarazada).	Fact 2: The progestin implant (Nexplanon) is very safe for people with health problems. The implant is a thin rod (about the size of a matchstick) that is placed in the upper arm after the skin has been numbed. Like IUDs, the implant can be used safely by almost everyone, even those with serious health problems. The implant has a 99% effectiveness rate and lasts up to 5 years. You can ask your healthcare provider to remove it at any time before then. The implant does not affect your fertility (your natural ability to get pregnant).	with regard to "before then" - These last 2 words are added because in the Spanish version is says "antes" at the end
**High Blood Pressure**<br>Your birth control options will depend on your blood pressure control and other risk factors.<br>High Blood Pressure during Pregnancy Only<br>This info is for people who had high blood pressure during pregnancy but now have normal blood pressure and do not take blood pressure medicines.<br>The good news is that you can use any type of birth control. This includes methods with and without hormones.<br>If you still have high blood pressure, read below.	**Presión Arterial Alta**<br>Tus opciones de control de la natalidad dependerán del control de tu presión arterial y de otros factores de riesgo.<br>Presión Arterial Alta solo durante el Embarazo<br>Esta información es para personas que tuvieron presión arterial alta durante el embarazo pero que ahora tienen la presión normal y no toman medicamentos para la presión arterial.<br>La buena noticia es que puedes usar cualquier tipo de método anticonceptivo. Esto incluye métodos con y sin hormonas.<br>Si todavía tienes presión arterial alta, lee a continuación.	**High Blood Pressure**<br>Your birth control options will depend on your blood pressure control and other risk factors.<br>High Blood Pressure only during Pregnancy<br>This information is for people who had high blood pressure during pregnancy but now have normal blood pressure and do not take blood pressure medication.<br>The good news is that you can use any type of contraception method. This includes methods with and without hormones.<br>If you still have high blood pressure, read on.	
**Diabetes**<br>Your birth control choices will depend upon whether diabetes has caused organ damage and how long you have had diabetes.<br>Diabetes can cause organ damage to the kidneys, eyes, nerves, and blood vessels over years. Medical terms for this organ damage include nephropathy, retinopathy, neuropathy, or arteriosclerosis (stiff arteries).<br>Special tests (blood, urine, eye exam, foot exam) can find organ damage early.	**Diabetes**<br>Sus opciones de anticonceptivos dependerán de si la diabetes ha causado daño a algún órgano y de cuánto tiempo ha tenido diabetes.<br>La diabetes puede causar daño a los órganos como a los riñones, los ojos, los nervios y los vasos sanguíneos a lo largo de los años. Los términos médicos para este daño orgánico incluyen nefropatía, retinopatía, neuropatía o arteriosclerosis (arterias endurecidas). Se puede detectar el daño a los órganos de manera temprana mediante pruebas especiales (sangre, orina, examen de los ojos, examen de los pies).	**Diabetes**<br>Your birth control options will depend on whether diabetes has caused organ damage and how long you have had diabetes.<br>Diabetes can cause damage to organs such as the kidneys, eyes, nerves, and blood vessels over the years. The medical terms for this organ damage include nephropathy, retinopathy, neuropathy, or arteriosclerosis (hardened arteries). Organ damage can be detected early through special tests (blood, urine, eye exam, foot exam).	
