# Supplementary material for: Translation Validation, Software Adaptation, and Usability Testing of a Spanish Contraception Decision Tool: Protocol for a Mixed Methods Pilot Study
Source: JMIR Res Protoc. 2026 Sep 30;15:e101800. doi: 10.2196/101800 (PMC13626405; doi:10.2196/101800)
Supplement: Supplementary file 2 [file resprot-v15-e101800-s001.docx]

To meet eligibility, patients must have at least one of the listed index health conditions and/or take at least one of the listed medications. Medications were chosen for their potential teratogenic properties and/or interactions with hormonal contraceptive methods.

**Index Health Conditions**

1. Acne Vulgaris (Active treatment)
2. Asthma (Active treatment)
3. Attention Deficit Hyperactivity Disorder (ADHD)
4. Bariatric Surgery (gastric sleeve, banding, bypass)
5. Breast Cancer
6. Breast Cysts, Benign Breast Disease
7. Cervical Cancer
8. Cervical Precancer (Dysplasia)
9. Chronic Pain Syndrome
10. Chronic Renal Disease
11. Crohn’s Disease
12. Cystic Fibrosis
13. Deep Venous Thromboembolism, Pulmonary Embolism (DVT, PE)
14. Depression, Bipolar, and Other Mood Disorders
15. Diabetes
16. Dialysis Use (Hemodialysis or Peritoneal)
17. Endometriosis
18. Gallbladder Problems
19. Gender affirming hormone therapy
20. Generalized Anxiety Disorder
21. Gestational Hypertension
22. Heart Disease, Heart Failure
23. Hepatic Disease (Fatty liver, alcohol, drug, or autoimmune causes)
24. Hepatic Tumors
25. Hepatitis B or C
26. Higher Body Weight (Overweight, Obesity)
27. Human Immunodeficiency Virus (HIV)
28. Human Papilloma Virus (HPV) infection
29. Hypercoagulable Disorders (Factor V Lieden, Antiphospholipid Antibody Syndrome, Factor C or S deficiency, prothrombin mutation)
30. Hyperlipidemia
31. Hypertension
32. Iron Deficiency Anemia (Active treatment)
33. Migraines With or Without Aura
34. Multiple Sclerosis
35. Ovarian Cancer
36. Ovarian Cysts or Benign Ovarian Tumors
37. Polycystic Ovary Syndrome (PCOS)
38. Peripartum Cardiomyopathy
39. Postpartum Depression
40. Premenstrual Dysphoric Disorder (PMDD)
41. Premenstrual Syndrome (PMS)
42. Pulmonary Arterial Hypertension
43. Rheumatoid Arthritis
44. Seizures
45. Sexually Transmitted Infections (Current or Past)
46. Sickle Cell Disease
47. Solid Organ Transplantation
48. Stroke
49. Substance Use Disorder
50. Superficial Venous Thromboembolism
51. Systemic Lupus Erythematosus (SLE)
52. Thalessemia
53. Thrombocytopenia
54. Tuberculosis
55. Thyroid Disorders
56. Ulcerative Colitis
57. Uterine Cancer
58. Uterine Fibroids
59. Uterine Hyperplasia
60. Valvular Heart Disease

**Characteristics**

1. Postpartum status
2. Smoking status
3. Breastfeeding status
4. Age
5. Weight/Body Mass Index (BMI)
6. Mobility limitations

**Medications**

*ACE inhibitors*

- Benzepril (Lotensin)
- Candesartan (Atacand)
- Captopril (Capoten)
- Enalapril (Vasotec)
- Eprosartan (Teveten)
- Irbesartan (Avapro)
- Lisinopril (Prinivil, Zestril)
- Losartan (Cozaar)
- Olmesartan (Benicar)
- Ramipril (Altace)
- Quniapril (Accupril)
- Valsartan (Diovan)
- Telmisartan (Micardis)

*Anticoagulants, Antiplatelets*

- Apixaban (Eliquis)
- Dabigatran (Pradaxa)
- Edoxaban (Savaysa)
- Rivaroxaban (Xarelto)
- Warfarin (Coumadin)

*Neuromodulators and Barbitals*

- A 'barbital' (Floricet, Florina, Fortabs, Esgic)
- Carbamazepine (Tegretol)
- Fosamprenevir
- Lamotrigine (Lamictal)
- Oxycarbazepine (Trileptal)
- Phenytoin (Dilantin)
- Primidone (Mysoline)
- Topiramate (Topamax, Qysmia)

*Statins*

- Atorvastatin (Lipitor)
- Fluvastatin (Lescor)
- Lovastatin (Mevacor, Altoprev)
- Pitavastatin (Livalo)
- Pravastatin (Pravachol)
- Rosuvastatin (Crestor)
- Simvastatin (Zocor)

*Other*

- Ambrisentan (Letairis)
- Bosentan (Tracleer)
- Doxycycline (Doryx, Vibramycine)
- Efavrienz (Sustiva)
- Isotretinoin (Accutane)
- Leflunomide (Arava)
- Lithium (Lithobid)
- Macitentan (Opsumit)
- Methimazole (Tapazole)
- Methotrexate (Trexall)
- Mycophenolate (Cellcept)
- Phenytoin (Dilantin)
- Phentermine/Topiramate (Qsymia)
- Testosterone
- Tetracycline
- Topiramate (Topamax)
- Valproic acid (Depakote)
