# Supplementary material for: Translation Validation, Software Adaptation, and Usability Testing of a Spanish Contraception Decision Tool: Protocol for a Mixed Methods Pilot Study
Source: JMIR Res Protoc. 2026 Sep 30;15:e101800. doi: 10.2196/101800 (PMC13626405; doi:10.2196/101800)
Supplement: Supplementary file 4 [file resprot-v15-e101800-s003.docx]

*System Usability Scale (SUS) – Modified for Use with Spanish-speaking users of Mi salud, mi decisión*

| No. | Pregunta | Scale | | | | |
| --- | --- | --- | --- | --- | --- | --- |
|  |  | Strongly  Disagree  [Totalmente en desacuerdo] |  |  |  | Strongly Agree  [Totalmente de acuerdo] |
| 1 | Pienso que el uso esta herramienta podría ayudarme a elegir el método anticonceptivo indicado para mi. (I think that I would use this tool to help me choose a birth control method that’s best for me) | 1 | 2 | 3 | 4 | 5 |
| 2 | Considero que esta herramienta fue muy complicado. (I thought the tool was too complicated) | 1 | 2 | 3 | 4 | 5 |
| 3 | Considero que esta herramienta es fácil de usar. (I thought the tool was easy to use) | 1 | 2 | 3 | 4 | 5 |
| 4 | Considero necesario el apoyo de alguien para usar esta herramienta. (I think that I would need the help of someone else to use the tool) | 1 | 2 | 3 | 4 | 5 |
| 5 | Considero que las diferentes partes de esta herramienta trabajan bien juntas. (I think the different parts of the tool worked well together) | 1 | 2 | 3 | 4 | 5 |
| 6 | Pienso que esta herramienta no siempre es clara y sus funciones no se alinean. (I felt like the tool wasn’t always clear and parts didn’t match up) | 1 | 2 | 3 | 4 | 5 |
| 7 | Considero que la mayoría de las personas aprenderían a usar esta herramienta rápidamente (I think most people would learn to use the tool very quickly) | 1 | 2 | 3 | 4 | 5 |
| 8 | Considero que toma mucho esfuerzo el uso esta herramienta. (I think the tool took too much effort to use) | 1 | 2 | 3 | 4 | 5 |
| 9 | Me sentí muy seguro al usar esta herramienta (I felt very confident using the tool) | 1 | 2 | 3 | 4 | 5 |
| 10 | Necesité aprender muchas cosas antes de empezar a usar esta herramienta. (I needed to learn a lot of things before I start using the tool) | 1 | 2 | 3 | 4 | 5 |
